# The need for socket preservation: a systematic review

**DOI:** 10.25122/jml-2021-0308

**Published:** 2022-03

**Authors:** Adel Alenazi, Abdulrahman Abdullah Alotaibi, Yazaid Aljaeidi, Nasser Raqe Alqhtani

**Affiliations:** 1. Department of Oral and Maxillofacial Surgery and Diagnostic Science, College of Dentistry, Prince Sattam Bin Abdulaziz University, Al-Kharj, Saudi Arabia

**Keywords:** implant, randomized controlled trial, socket, tooth extraction

## Abstract

The aim of this study was to evaluate the clinical need and impact of socket preservation to protect the bone for future dental implant placement. Moreover, we aimed to list down various methods of socket preservation by going through randomized clinical trials. We searched PubMed, Google Scholar, and Cochrane databases for all relevant publications, where researchers compared various methods and tools for socket preservation. All eight randomized controlled trials mentioned several methods that are helpful in preserving bone levels both horizontally and vertically. The studies included in this systematic review demonstrate that each material has certain efficacy in preserving the socket after tooth extraction for future implant placement. Socket preservation methods and materials are effective in preparing patients for future prostheses.

## INTRODUCTION

Tooth extraction affects masticatory efficiency and causes homeostatic and structural changes in periodontal tissues, leading to alveolar ridge atrophy. Alveolar ridge preservation (ARP) is carried out to avoid ridge resorption after extraction [1, 2]. In the past two decades, many treatment choices were mentioned, such as socket grafting with a biomaterial alone interposing a barrier element. However, there is no resolution regarding the best method for socket preservation: autogenous, allogenic, or alloplastic [2]. Conserving the alveolar ridge is effective but technically delicate, requiring specific surgical skills [1–3]. Still, there is insufficient proof regarding the success of these techniques and the advantages of one method over the other. Presently conflicting observations are reported by researchers regarding the use of grafting material for ARP to prevent alveolar ridge resorption [2, 4, 5].

Our research question aims to identify the effect of various socket preservation materials and methods on the maintenance of ridge levels among patients requiring future prostheses.

This systematic review was carried out to evaluate the clinical need for socket preservation to preserve bone for future placement of a dental implant. Moreover, we aimed to list down various methods of socket preservation by going through randomized clinical trials (RCT). The target audience of this systematic review includes periodontists as well as prosthodontists.

## MATERIAL AND METHODS

PubMed, Google Scholar, and Cochrane were searched for all relevant publications where the researchers compared various methods and tools for socket preservation. The keywords used were “socket preservation”, “alveolar ridge preservation”, and “bone grafts”. The following search resulted in a total of 76 citations found. After adjusting for duplicates, 72 publications remained. Next, the abstracts of the articles were reviewed, after which 64 studies were removed.

*Inclusion criteria:* The following criteria were considered essential for the inclusion into the systematic review: (1) randomized control trials, (2) human studies, (3) included the previously mentioned keywords, (4) English language of publication, (5) trials focusing on socket preservation methods and results.

*Exclusion criteria:* (1) case-control studies, (2) cross-sectional studies, (3) article language other than English, (4) *in vitro* studies, (5) cohort studies, (6) animal studies.

**Figure 1.**
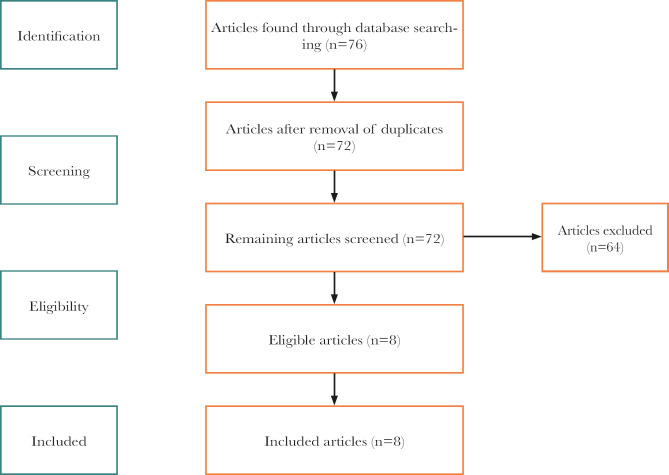
PRISMA flowchart.

We used the PRISMA flowchart to report the information received during the examination. Selçuk (2019) [5] highlights that PRISMA (Preferred Reporting Items for Systematic Reviews and Meta-Analyses) is used to improve transparency in systematic reviews. Therefore, this systematic review adhered to the PRISMA guidelines to eliminate bias and ensure successful completion. Figure 1 represents the PRISMA chart demonstrating various phases of the systematic review.

## RESULTS

All studies included (n=8) were thoroughly reviewed, and their findings will be presented in detail in this section. The first study discussed was performed by Avila-Ortiz *et al.* [1], who completed alveolar ridge preservation using bone allografts and non-absorbable membrane *vs.* extraction only. Their findings suggest that the ARP method was better than the extraction-only option. As far as the linear bone outcomes were concerned, a mean horizontal crestal width change of 1.07 mm (95% CI, −1.49 to −0.64; P<0.0001). In the same way, there was a noteworthy change in ΔBRH (Buccal Ridge Height) as the median ΔBRH was 0.61 mm (IQR, 0.46 to 0.94) in ARP.

The second study [6] used a full thickness mucoperiosteal flap and a flapless procedure. The results revealed that the changes of the buccolingual bone width were 3.5±0.9 mm for the flap group and 1.7±0.6 mm for the flapless group (p<0.001). In what concerns the vertical bone (VB), the statistical analysis showed only one significant comparison (p=.0105) for VB in the flapless group (1.1±0.9 mm), whereas, in the flapped group, the value obtained for VB was 0.6±0.7 mm. The third study was conducted by Meloni *et al.* [7], who suggested that the epithelial connective tissue graft led to 0.26 mm bone loss vertically and 1.60 mm horizontally. The porcine collagen matrix led to a vertical loss of 0.31 mm bone and 1.47 mm horizontally.

The study conducted by Guarnieri *et al.* [8] used three techniques, including extraction sockets with spontaneous bleeding (S), extraction sockets covered by collagen membrane alone (M), and extraction sockets grafted with porcine-derived bone (GM). Results showed that the S group observed 2.13 mm vertically and 3.96 mm horizontally; the M group observed 0.58 mm vertically and 0.91 mm horizontally; the GM group observed 0.31 mm vertically and 0.91 mm horizontally.

Maiorana *et al.* [9] used demineralized bovine bone mineral and covered it with a porcine-derived non-crosslinked collagen matrix. The findings revealed resorption of 1.21 mm horizontally and 0.46 mm vertically.

Machtei *et al.* [10] used biphasic calcium sulfate/hydroxyapatite (BCS/HA); bovine-derived xenograft (BDX), or no grafting (control group). Their results showed resorption of 0.65 mm in BCS/HA, 0.25 mm in BDX, and 1.71 mm in the control group were observed vertically. At the same time, 0.5 mm in BCS/HA, 1.56 mm in BDX, and 6 mm in the control group were observed horizontally.

The study by de Carvalho Formiga *et al.* [11] used dense PTFE membranes with and without xenograft material. Results revealed changes in the buccal plate: control group 0.46 mm, test group 0.91 mm; and alveolar height: control group −0.41 mm, test group 0.35 mm were observed.

Finally, the study by Cardaropoli *et al.* [12] used extraction alone *vs.* bovine bone mineral and collagen membrane. Findings revealed that 1.04 mm (width) and 0.46 mm (height) were observed in the experiment group. In contrast, 4.48 mm (width) and 1.54 mm (height) were observed in the extraction alone group. The summary of the studies is described in Table 1.

**Table 1.** Summary of the studies included in the systematic review.

NO	Article (Reference)	Inclusion criteria	Methodology	Results and findings
**1.**	Avila-Ortiz *et al*. [1]	RCT, human studies, socket preservation methods, and results	Alveolar Ridge Preservation (ARP) using bone allograft, non-absorbable membrane *vs*. only tooth extraction	Horizontal crestal width change: -1.07mm Median ΔBRH was 0.61 mm Median ΔLRH was 0.47 mm
**2.**	Barone *et al*. [6]	RCT, human studies, socket preservation methods, and results	Full-thickness mucoperiosteal flap *vs*. a flapless procedure	Epithelial connective tissue graft: Vertical bone loss: 0.26 mm Horizontal: 1.60 mm Porcine collagen matrix: Vertical: 0.31 mm Horizontal: 1.47 mm
**3.**	Meloni *et al*. [7]	RCT, human studies, socket preservation methods, and results	Epithelial connective tissue graft *vs*. porcine collagen matrix	Epithelial connective tissue graft: Vertical bone loss: 0.26 mm Horizontal: 1.60 mm Porcine collagen matrix: Vertical: 0.31 mm Horizontal: 1.47 mm
**4.**	Guarnieri *et al*. [8]	RCT, human studies, socket preservation methods, and results	Porcine-derived collagen membrane *vs*. natural spontaneous healing	Extraction sockets with spontaneous bleeding (S): -2.13 mm vertically and -3.96 mm horizontally Extraction sockets covered by collagen membrane alone (M): -0.58 mm vertically and -0.91 mm horizontally Extraction sockets grafted with porcine-derived bone (GM): -0.31 mm vertically and -0.91 mm horizontally.
**5.**	Maiorana *et al*. [9]	RCT, human studies, socket preservation methods, and results	Demineralized bovine bone mineral covered with a porcine-derived non-crosslinked collagen matrix	1.21 mm horizontally 0.46 mm vertically.
**6.**	Machtei *et al*. [10]	RCT, human studies, socket preservation methods, and results	Biphasic calcium sulfate/hydroxyapatite bovine-derived xenograft	0.65 mm in BCS/HA, 0.25 mm in BDX, and 1.71 mm in the control group (vertically). 0.5 mm in BCS/HA, 1.56 mm in BDX, and 6 mm in the control group were observed horizontally.
**7.**	de Carvalho Formiga M *et al*. [11]	RCT, human studies, socket preservation methods, and results	Bone graft *vs*. blood clots	Buccal plate: control group 0.46 mm, test group 0.91 mm; Alveolar height: control group −0.41 mm, test group 0.35 mm
**8.**	Cardaropoli *et al*. [12]	RCT, human studies, socket preservation methods, and results	Extraction alone *vs*. bovine bone mineral	Experiment group: 1.04 mm (width) and 0.46 mm (height) Extraction alone group: 4.48 mm (width) and 1.54 mm (height)

### Quality assessment

The Cochrane risk of bias assessment method was used to assess the quality of the studies included (Table 2).

**Table 2.** Cochrane Risk of bias assessment.

	Random sequence generation	Allocation concealment	Participant and personnel blinding	Outcome Assessment Blinding	Incomplete outcome data	Selective reporting	Other bias
**Avila-Ortiz *et al.*, (2020) [1]**							
**Barone *et al.*, (2014) [6]**							
**Meloni *et al.*, (2015) [7]**							
**Guarnieri *et al.*, (2017) [8]**							
**Maiorana *et al.*, (2017) [9]**							
**Machtei *et al.*, (2019) [10]**							
**De Carvalho Formiga *et al.*, (2019) [11]**							
**Cardaropoli *et al.*, 2012 [12]**							
					Low	Unclear	High

## DISCUSSION

This study aimed to list various methods and materials used to preserve the sockets and report the effectiveness of materials and methods discussed. It can be noted from the findings that almost all the methods mentioned produced positive outcomes, especially when the authors compared the findings of the experimental group with the control group. In addition, it was noted that methods such as non-absorbable membrane (dPTFE) and porcine-derived collagen membrane showed positive outcomes when the horizontal bone loss was measured. However, other mentioned materials and methods were effective in preserving vertical bone loss, but not when it comes to horizontal bone loss preservation [9–12]. RCTs using xenografts displayed a considerable diminution of the alveolar bone [1, 7, 13]. It was shown that even the most careful extraction bone resorption results necessitate bone augmentation [14].

## CONCLUSIONS

The studies included in this systematic review demonstrate that each material has a certain amount of efficacy in preserving the socket. The methods mentioned above can be used to provide adequate bone preservation both horizontally and vertically, considering the needs of each patient and cost-bearing capability.

## ACKNOWLEDGMENTS

### Conflict of interest

The authors declare no conflict of interest.

### Authorship

AA contributed to conceptualizing and methodology. AAA contributed to methodology and data collection. YA contributed to writing the draft, and NRA contributed to the literature search and data analysis.
